# Haemophagocytic lymphohistiocytosis due to *Burkholderia pseudomallei* in a primigravida

**DOI:** 10.1099/acmi.0.000520.v3

**Published:** 2023-09-13

**Authors:** Shaikh Mohammed Haroon al Waseem, Tessa Antony, Suchitra Suresh, Sowmya Gopalan

**Affiliations:** ^1^​ Past postgraduate, Department of Microbiology, Sri Ramachandra Medical College and Research Institute, Chennai, India; ^2^​ Department of Microbiology, Sri Ramachandra Medical College and Research Institute, Chennai, India; ^3^​ Department of General Medicine, Sri Ramachandra Medical College and Research Institute, Chennai, India

**Keywords:** blood culture, *Burkholderia pseudomallei*, haemophagocytic lymphohistiocytosis

## Abstract

**Introduction.:**

Melioidosis is caused by *Burkholderia pseudomallei,* a Gram-negative, saprophytic bacillus, commonly found in soil or contaminated water. As infection with this bacterium produces a wide variety of clinical manifestations the organism is aptly called the ‘great mimicker’. Even though it is non-fastidious and an easily cultivable organism, it can be misidentified in automated identification systems.

**Case report.:**

A 24-year-old primigravida presented with complaints of fever and myalgia of 45 days’ duration. She was diagnosed to have haemophagocytic lymphohistiocytosis (HLH) based on clinical and laboratory parameters. Blood and bone marrow culture sent to the microbiology laboratory grew non-fermenting Gram-negative bacilli which were misidentified as *Burkholderia cepacia* by matrix-assisted laser desorption ionization-time of flight mass spectrometry (MALDI-TOF MS) technology. It was subsequently identified as *B. pseudomallei* by 16S rRNA gene sequencing. The patient was commenced on intensive phase therapy with intravenous ceftazidime for 2 weeks, followed by maintenance therapy with oral trimethoprim and sulfamethoxazole for 3 months. In view of HLH, she was treated with intravenous dexamethasone for 2 weeks which was later switched to oral dexamethasone for a period of 6 weeks. She responded well to the treatment, but had to undergo medical termination of her pregnancy as there was severe intrauterine growth restriction of the fetus.

**Conclusion.:**

Prognosis of melioidosis is excellent if early diagnosis and appropriate antibiotic treatment is provided. In this era of automation, it is important to determine if the suspected pathogen is listed in the database of the automated identification system.

## Data Summary

No data were generated as this is a case report.

## Introduction

Haemophagocytic lymphohistiocytosis (HLH) is a severe systemic inflammatory syndrome that can result in deregulated engulfment of haematopoietic cells by lymphocytes and activated macrophages. HLH may be inherited in an autosomal recessive manner or it can be acquired following various infections, malignancy, metabolic or rheumatological conditions, and is otherwise called secondary HLH. The development of secondary HLH in infections may be due to an inability to suppress or clear intracellular infections resulting in persistent activation of antigen presenting cells and CD8(+) T cells and increased production of cytokines. Secondary HLH is a potentially fatal complication of *Burkholderia cepacia* infection and has been reported in patients with chronic granulomatous disease [1, 2]. There are very few reported cases of HLH due to *Burkholderia pseudomallei* [3]. As *B. pseudomallei* is a non-fastidious bacterium, it can be easily isolated from clinical samples, but can be misidentified in automated systems. We report a case of HLH due to *B. pseudomallei,* a facultative intracellular pathogen.

## Case report

A 24-year-old primigravida was referred to the medicine department of the hospital with complaints of intermittent high-grade fever associated with chills and generalized body pain for the past 45 days. There was no other significant medical history. The patient was a home maker living in a coastal city in southern India. She had been evaluated as an inpatient for the current illness in two other healthcare facilities, with no conclusive diagnosis, before the present hospital admission.

Physical examination revealed the patient to be febrile with appreciable pallor. Per abdomen uterus size corresponded to 22 weeks of gestation. Examinations of other systems were unremarkable. Laboratory investigations revealed anaemia (haemoglobin: 7.3 g dl^−1^, total white blood cell count: 9290 cells mm^−3^, platelet count: 3.17×10^5^ mm^−3^). Biochemical markers demonstrated a raised triglyceride level (3.1751 mmol l^−1^) and serum ferritin levels (1619 µg l^−1^) and normal liver function. Bone marrow biopsy showed reduced normoblastic maturation of all lineages, presence of haemophagocytes, normal granulopoiesis and normal megakaryocytes with absent iron stores.

Screening tests for autoimmune markers (rheumatoid arthritis factor, antinuclear/cytoplasmic antibodies) were negative. 2D echo showed no signs of infective endocarditis. Magnetic resonance imaging (MRI) of the thorax was normal study. MRI of the abdomen revealed hepatosplenomegaly and a single live intrauterine gestation. The patient was diagnosed to have HLH, as she had five of the eight criteria needed for diagnosis, which included presence of fever, splenomegaly, haemophagocytes in bone marrow, raised serum ferritin and raised triglyceride levels. Detection of soluble IL-2 receptor levels and natural killer cell activity could not be done in the patient as these tests are not performed routinely in the laboratory.

Blood culture showed growth of non-fermenting Gram-negative bacilli which were presumptively identified as *Acinetobacter* species according to conventional biochemical tests but were found to be resistant to polymyxin B discs (300 units).

**Fig. 1.**
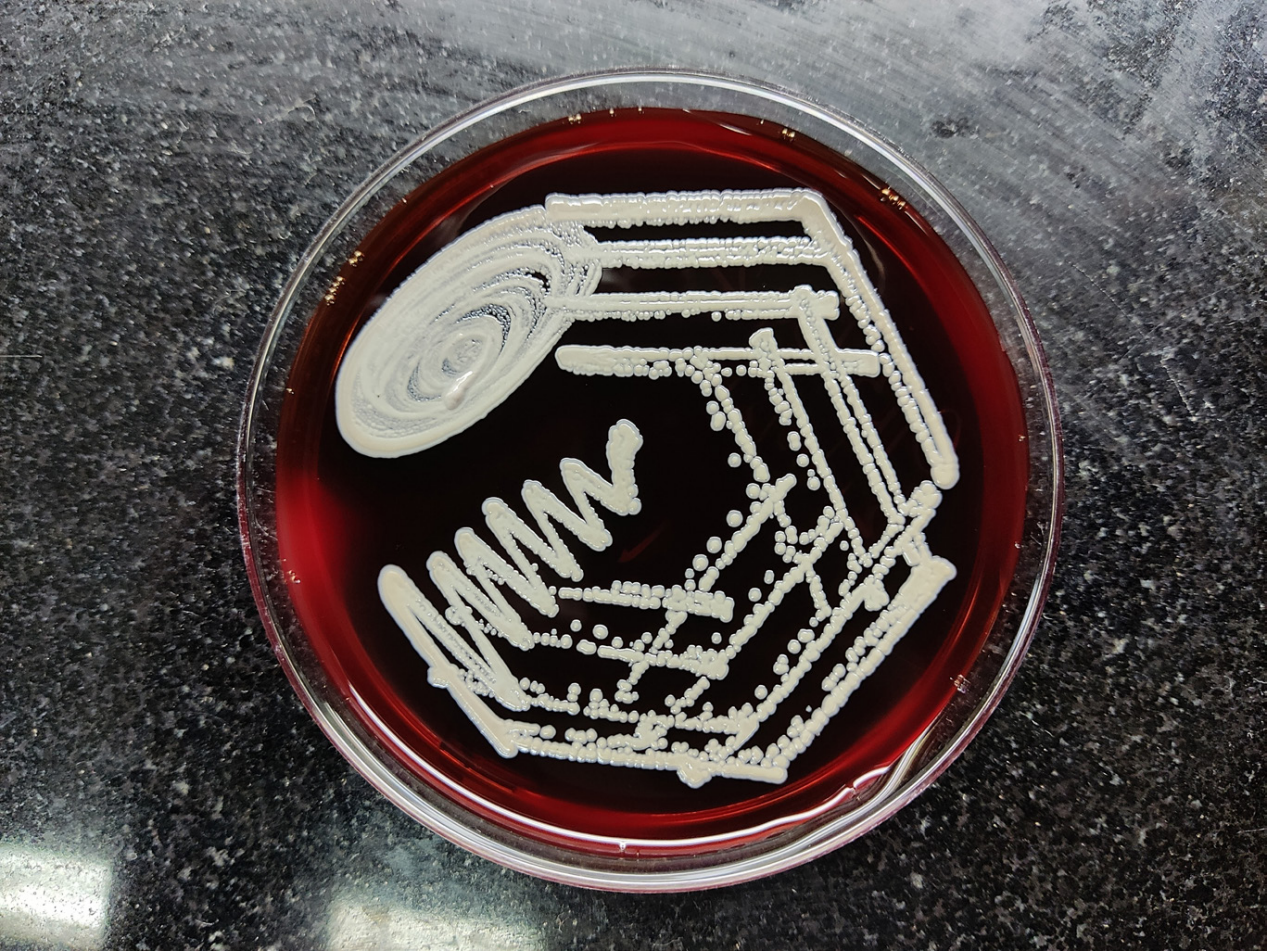
Colonies of *B. pseudomallei* on 5 % sheep blood agar (day 4).

**Fig. 2.**
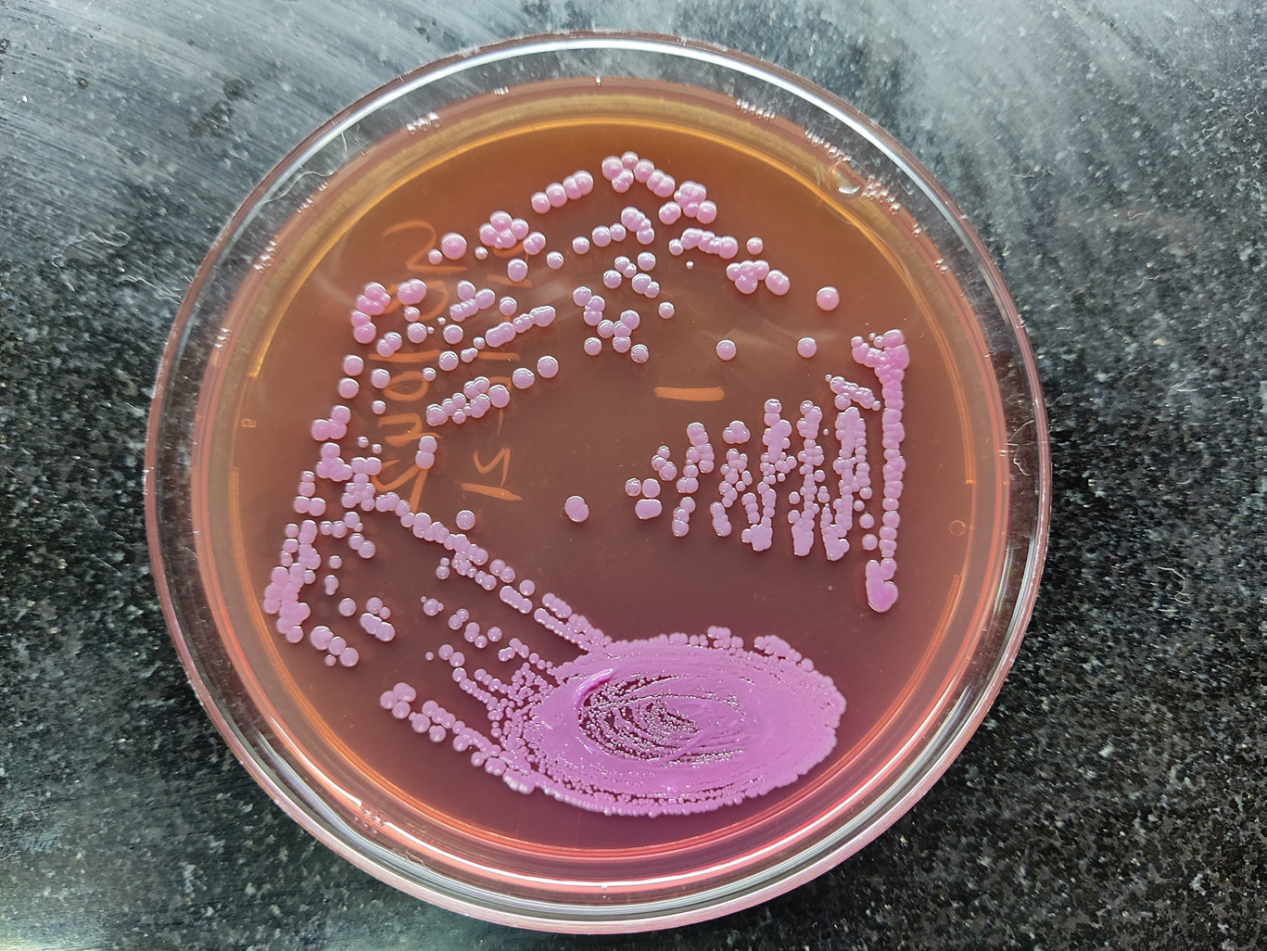
Colonies of *B. pseudomallei* on MacConkey agar (day 4).

Blood and bone marrow samples were sent again for bacterial culture after 1 week. Colonies of non-fermenting Gram-negative bacilli grew on 5 % sheep blood agar (Fig. 1) and MacConkey agar (Fig. 2). Even though the bacteria did not ferment lactose, the colonies on MacConkey agar appeared pink after a few days due to uptake of the dye from the medium. It was found to be delayed oxidase-positive and was identified as *B. cepacia* with 30 % probability by matrix-assisted laser desorption ionization-time of flight MS (MALDI-TOF MS) (VITEK MS; bioMérieux). We further found that *B. pseudomallei* was not present in the database of the VITEK MS system. Further identification to the species level was done by PCR-based DNA sequencing of the 16S rRNA gene (GenBank accession number OP764052) and growth of *B. pseudomallei* was reported. It was found to be susceptible to ceftazidime, imipenem, meropenem, trimethoprim/sulfamethoxazole, amoxicillin/clavulanic acid and tetracycline. Antimicrobial susceptibility testing was done using the automated Vitek 2 COMPACT system (bioMérieux) with AST-N406 panel for Gram-negative bacilli, and the break points (MICs) were interpreted according to M45 CLSI (Clinical Laboratory Standards Institute) Guidelines 2018. Currently there are disc diffusion guidelines in EUCAST for interpreting susceptibility of *B. pseudomallei* to antimicrobial agents.

The patient was initially started on intravenous piperacillin tazobactam, as per the blood culture susceptibility report of *Acinetobacter* species, which was then changed to intravenous ceftazidime 1 g 6-hourly for 2 weeks. In view of HLH, she was given intravenous dexamethasone 8 mg twice daily for 2 weeks, followed by oral dexamethasone 4 mg twice daily which was tapered and stopped over a period of 6 weeks. She received three units of packed cell transfusion as well as parenteral supplementation of iron for correction of anaemia.

The patient was counselled for termination of her pregnancy as there was severe intrauterine growth restriction (IUGR) of the fetus. Following stabilization of the patient’s condition, medical termination of the pregnancy was done. The patient was symptomatically better and her fever resolved within 72 h of initiation of therapy . Considerable improvements in the clinical and laboratory parameters were observed in the first week of treatment. Hence, the intensive therapy was followed by maintenance therapy of oral trimethoprim/sulfamethoxazole 240 mg/1200 mg twice a day over a period of 3 months. Repeat haemoglobin testing 2 weeks later showed a raised haemoglobin level of 11.9 g dl^−1^.

## Discussion

Melioidosis is endemic in northern Australia and Southeast Asian countries, especially Thailand and Malaysia, and is being increasingly recognized in other regions. The global incidence of human melioidosis is approximately 165 000 cases per year worldwide and South Asia has the highest burden of the disease (44 % of all cases). As the largest country in South Asia with a large diabetic population and favourable environmental conditions, India is a potential hotspot for the disease [4].

According to the updated Henter criteria of 2004, a diagnosis of HLH can be made if five of the following eight criteria are satisfied, including the presence of fever, splenomegaly, cytopenias affecting two or more of three cell lineages, hypertriglyceridaemia and/or hypofibrinogenaemia [fasting triglyceride ≥3 mmol l^−1^; fibrinogen (≤1.5 g l^−1^)], haemophagocytosis, hyperferritinaemia (≥500 µg l^−1^), high soluble IL-2 receptor levels (≥2400 U ml^−1^), and reduced or absent natural killer cell activity [5]. The list of infectious aetiologies for secondary HLH includes viruses (e.g. *Epstein-Barr virus, Herpes simplex virus, Cytomegalovirus, avian Influenza virus*), bacteria (*Mycobacterium tuberculosis, Borrelia, Brucella, Coxiella burnetii,* Leptospira) and fungi (Candida*, Cryptococcus neoformans*) [6–8].

The patient in the present case report did not have any history of chronic granulomatous disease or cystic fibrosis nor did she undergo any invasive procedure or prolonged hospitalization for acquisition of *B. cepacia* infection. In this scenario, we sent the bacterial isolate for further identification by 16S rRNA gene sequencing. This case highlights that *B. pseudomallei* could be another possible causative bacterial agent of HLH.

Individuals with diabetes mellitus, renal disease or thalassaemia, or those undergoing immunosuppressive therapy and those with alcohol misuse are at increased risk of acquiring melioidosis. A review of local data in Malaysia showed that *B. pseudomallei* infection can occur in 15–42 % of individuals even in the absence of these risk factors [9]. The patient mentioned in this case report was a young, immunocompetent primigravida. There is no clear evidence on antenatal outcomes with melioidosis, and it is not known if pregnancy is a risk factor for *B. pseudomallei* infection or if such individuals experience more severe disease [10].

A definitive diagnosis of melioidosis requires a positive culture of *B. pseudomallei *. The organism can be misidentified as *Pseudomonas* spp. or *Acinetobacter* spp. if a detailed biochemical analysis is not performed. Commercially available identification systems and automated identification algorithms may misidentify *B. pseudomallei* as other *Burkholderia* spp. such as *B. cepacia* complex, *Chromobacterium violaceum* and *Pseudomonas* spp. However, the pattern of resistance to antimicrobials is distinctive as most *B. pseudomallei* isolates are resistant to aminoglycosides and polymyxin B .

Molecular methods such as 16S rRNA gene sequencing are widely used for identification of bacteria to the species level in clinical microbiology laboratories and are applicable to *B. pseudomallei* also. Other gene targets used for identification of *B. pseudomallei* include *groEL* and the type III secretion system [11].

Detection of specific antibodies can be done in cases of chronic melioidosis, but acutely bacteraemic patients appear less likely to have antibodies [11]. Antigen detection tests can be done directly on clinical specimens (e.g. sputum, urine or pus), but the sensitivity is significantly lower than with culture [12].

Treatment for melioidosis includes two phases, an initial intensive phase and subsequent eradication phase. The intensive phase includes the use of intravenous ceftazidime and carbapenem (either meropenem or imipenem) for at least 10–14 days. The addition of trimethoprim sulfamethoxazole for a longer duration of the intensive phase is required for those patients who are critically ill or in patients with extensive pulmonary disease, organ abscesses, osteomyelitis, septic arthritis and neurological melioidosis. The eradication/maintenance phase is for a minimum of 3 months and the preferred drug is trimethoprim sulfamethoxazole [13]. Alternative drugs used in eradication therapy include amoxicillin-clavulanic acid, oral quinolones (ciprofloxacin, levofloxacin), doxycycline and chloramphenicol. Amoxicillin-clavulanic acid is preferred in pregnant women and in those who are intolerant of trimethoprim sulfamethoxazole. Standard therapy for HLH involves administration of steroids, cyclosporin and etoposide. Steroids are added to the antimicrobial agent when the bacterial cause for HLH has been identified.

## Conclusion

*B. pseudomallei* can be a rare cause of HLH. Early and accurate identification of the organism is essential for optimal treatment as it requires prolonged antibiotic therapy that includes an intensive phase and maintenance phase. Its important to ensure the suspected organism is present in the database of the automated identification system. Collaboration between the treating physician and the microbiologist is vital in improving the outcome in patients with HLH.
